# Highly Active Antiretroviral Therapy (HAART) - “Sulfonyl”, and anal cancer outcomes from patients living with HIV: a retrospective cohort

**DOI:** 10.1186/s13027-025-00666-y

**Published:** 2025-08-21

**Authors:** Raelson Rodrigues Miranda, Erika Andrade Rocha, Amanda Acioli de Almeida Robatto, Denis Artico Galhera, Carolina Ribeiro Victor, Karim Yaqub Imbrahim, Camila Motta Venchiarutti Moniz

**Affiliations:** 1 Instituto do Câncer do Estado de São Paulo, São Paulo, Brazil; 2 Pós-Graduação, Faculdade de Medicina da Universidade de São Paulo, São Paulo, Brazil; 3 Universidade Municipal de São Caetano do Sul, São Caetano do Sul, Brazil; 4 Department of Clinical Oncology, Instituto do Câncer do Estado de São Paulo, 251, Avenida Doutor Arnaldo, Cerqueira Cesar, São Paulo, 01246000 Brazil

**Keywords:** Anal Cancer, HighlyActive Antiretroviral Therapy, HIV, Radiotherapy, Chemotherapy

## Abstract

**Background:**

Although anal cancer is a rare malignancy, its incidence is up to 30 times higher among individuals living with HIV. Recent evidence suggests that Highly Active Antiretroviral Therapy (HAART) regimens containing sulfonyl groups may exhibit antitumor properties. Based on these findings, we hypothesize that HAART regimens incorporating sulfonyl-containing compounds could influence oncologic outcomes in HIV-positive patients undergoing definitive chemoradiotherapy (CRT) for anal cancer.

**Methods:**

From a cohort of 537 patients with stage I–III invasive anal cancer treated between August 2010 and April 2022, 56 HIV-positive patients who underwent definitive chemoradiotherapy were included in the analysis.

.

**Results:**

Most patients were male. The mean age was 52 years in the non-sulfonyl-HAART group and 53 years in the sulfonyl-HAART group. The mean CD4 count was significantly higher in the non-sulfonyl group compared to the sulfonyl group (523 vs. 287 cells/mm³, p = 0.02). Grade 3–4 toxicities occurred in 60% and 38% of patients, respectively (p = 0.18). Chemotherapy dose reductions were required in 10% of the non-sulfonyl group and 8% of the sulfonyl group (p = 1.0). Treatment discontinuation during chemoradiotherapy occurred in 17% vs. 23% of patients, respectively (p = 0.7). The overall response rate at 6 months post-treatment was significantly higher in the sulfonyl-HAART group (100%) compared to the non-sulfonyl group (20/36; 55.6%), Odds Ratio (OR) 0.00, 95% CI: 0–0.72, p = 0.004). After adjustment, CD4 count was not associated with treatment response (logistic regression OR: 1.00; 95% CI: 0.99– 1.00, p = 0.3). The median progression-free survival (PFS) in the non-sulfonyl-HAART group was 70 months (p = 0.45), and overall survival (OS) was similar between groups (p = 0.6); the median OS was not reached in the sulfonyl-HAART group. In the Cox proportional hazards model, age, clinical stage, and lack of response to CRT at 6 months were independent predictors of worse survival. (

**Conclusion:**

HIV-positive patients with anal cancer who received sulfonyl-containing HAART during definitive chemoradiotherapy demonstrated a significantly higher overall response rate at 6 months, independent of baseline CD4 count. However, no significant differences were observed between the sulfonyl and non-sulfonyl groups in terms of treatment-related toxicities, treatment discontinuation, progression-free survival, or overall survival.

## Introduction

Anal cancer is a rare solid tumor with an estimated incidence rate of around 2 cases for every 100.000 people in the USA, according to the National Cancer Institute. Most cases are diagnosed at localized stages, and the survival in 5 years is higher compared to the metastatic scenario: 84% versus 36% [1]. In HIV patients younger than 30 years, the cumulative incidence for men who have sex with other men is 0.17%, 0.04% for men, and 0.03% for women. This risk rises when men who have sex with other men are diagnosed with AIDS for 0.35% [2]. 

In localized disease scenario, radio and chemotherapy have been the backbone of the treatment of anal carcinoma for decades, even with some recent changes in the course of radiotherapy and the best combinations of chemotherapy [3]. On the other hand, Highly Active Antiretroviral Therapy (HAART) is the base of treatment for HIV patients. Some drugs, such as Zidovudine, can induce neutropenia in 8% of patients with AIDS. Other ones, such as didanosine and stavudine, can be associated with peripheral neuropathy, a side effect usually seen in some anticancer drugs [4]. Moreover, as with HAART, many chemotherapies can be metabolized by CYP450, and therefore, drug interactions between antiretrovirals and anticancer therapy have been the subject of review. Interactions between HAART and chemotherapies used for anal cancer, such as 5-FU and mitomycin, are not well-documented. However, cisplatin combined with tenofovir, ritonavir, and indinavir increases nephrotoxicity [4–7]. 

Sulfonyl, Sulfonamide, or sulfa, are chemical compounds derived from the sulfonic acid group (RSO3H) and show wide biological activities including diuretic, anti-diabetic, anti-bacterial, protease inhibitors, anti-fungal, and anti-cancer [8, 9]. Its use is widely explored by pharmaceuticals in developing drugs cause of their applications such as solubility modulation, improving the interaction between drugs and therapeutical targets, anti-inflammatory properties, and enzymatic inhibitors [9, 10]. Using cell lines, mice, and human cord blood researchers found in vitro that sulfonamide-derived InhiTinib induced apoptosis and T-cell responses [11]. In another study, sulfonyl derivates also showed a block of VEGFR-2 [10, 12]. 

As explained above, there are vast interactions between anti-cancer and anti-retroviral drugs. However, the interactions between these drugs during the treatment of radiotherapy and how they affect toxicity and survival outcomes are poorly explored. We hypothesized that anti-retroviral contenting sulfonyl derivates due to anti-cancer properties can increase side effects and survival. Here, we present clinical and survival outcomes from a retrospective cohort comparing groups of patients HIV positive with invasive anal carcinoma taking HAART with Sulfonyl-R and Non-Sulfonyl-R during chemoradiotherapy (CRT).

## Methods

### Design and ethical aspects

This is a retrospective cohort of patients with anal carcinoma and HIV-positive, which were treated with chemotherapy and radiotherapy as curative intention between August 2010 and April 2022 in a single center at Instituto do Câncer do Estado de São Paulo, Brazil. STROBE Guidelines (Equator Network) were used to write this manuscript, and to avoid plagiarism, we reviewed it on Viper plagiarism checker (Nottingham, UK).

### Definitions and procedures

The collection of epidemiologic data from patients’ records and anonymization was made using the RedCap platform (Vanderbilt University). Data collection included age, gender, race, histologic type, staging, type of radiotherapy, HIV status, CD4 + cell count, treatment toxicity, treatment discontinuation, type of HAART, recurrence of disease, and surgery rescue. Magnetic resonance imaging (MRI) of the pelvis was utilized to assess the response rate according to RECIST v1.1 criteria at 8 weeks and 6 months following the completion of concomitant chemoradiotherapy. Additionally, a digital rectal exam (DRE), proctoscopy, and biopsy were performed to evaluate the presence of a complete response. Patients with complete response were followed with DRE, anoscopy, and chest/abdomen/pelvis every 6 months for 3 years, and after that, annually. For patients with persistent disease, we performed re-imaging (CT scans) according to physician choice. In the cases of regression, we continued observation, but in cases of local progression or metastatic disease, surgery rescue or systemic treatment was provided. Progression-free survival was defined as the time between the end of chemoradiotherapy and recurrence or progression local, or occurrence of metastasis. Overall survival was defined as the time between the date of histopathologic diagnosis and the date of death or the date of last attendance at the hospital. For comparison of all variables, patients were divided between two groups: HAART containting sulfonyl-radical (Sulfonyl-HAART), and HAART without sulfonyl-radical (Non-sulfonyl-HAART). We have used PubChem (https://pubchem.ncbi.nlm.nih.gov/*)* to identify the IUPAC (International Union of Pure and Applied Chemistry) name of antiretrovirals, and search for ‘sulfonyl’ or ‘sulfonamide’. Drugs such as amprenavir, fosamprenavir, darunavir, and tipranavir were defined as HAART containting “sulfonyl”. Any use of a HAART combination containting those drugs during chemoradiotherapy was called the sulfonyl-HAART group. On the other hand, combinations without them were included in the non-sulfonyl-HAART group. The primary endpoint of this analysis was overall survival (OS). Secondary endpoints included progression-free survival (PFS), response rate, adverse events, toxicities grade 3 and 4, and rescue surgery rate.

### Sample size and statistical analysis

We performed a screening in the dataset of our institution looking for ‘anal cancer’, and ‘ICD-10: C21’ between Aug-2010 and Apr-2022 in stages I, II, and III (AJCC 8a ed.). Then, we searched for patients who were HIV-positive and were treated with definitive chemoradiotherapy at early setting. For all variables, we assumed an two-sided alpha error of 5%. We performed Kaplan-Meyer curves to summarise the overall and progression-free survival, and the Log-rank test was used to compare two survival rates between sulfonyl-HAART and non-sulfonyl-HAART groups. For univariate analysis, continuous variable without normal distribution we performed log transformation and used t-test. Fisher’s exact, chi-square, and Likelihood were used for categorical variables. Logistic regression was done to test the association force between variables of interest. Additionally, for the variables of interest or those had statistical significance in the univariate analysis, we proceeded with the multivariate analysis using the Cox proportional hazard to adjust covariates and identify the confounders and independent risk variables to survival outcomes. All the statistics assay was made using STATA v.17.

## Results

### Characteristics of patients

**Fig. 1:**
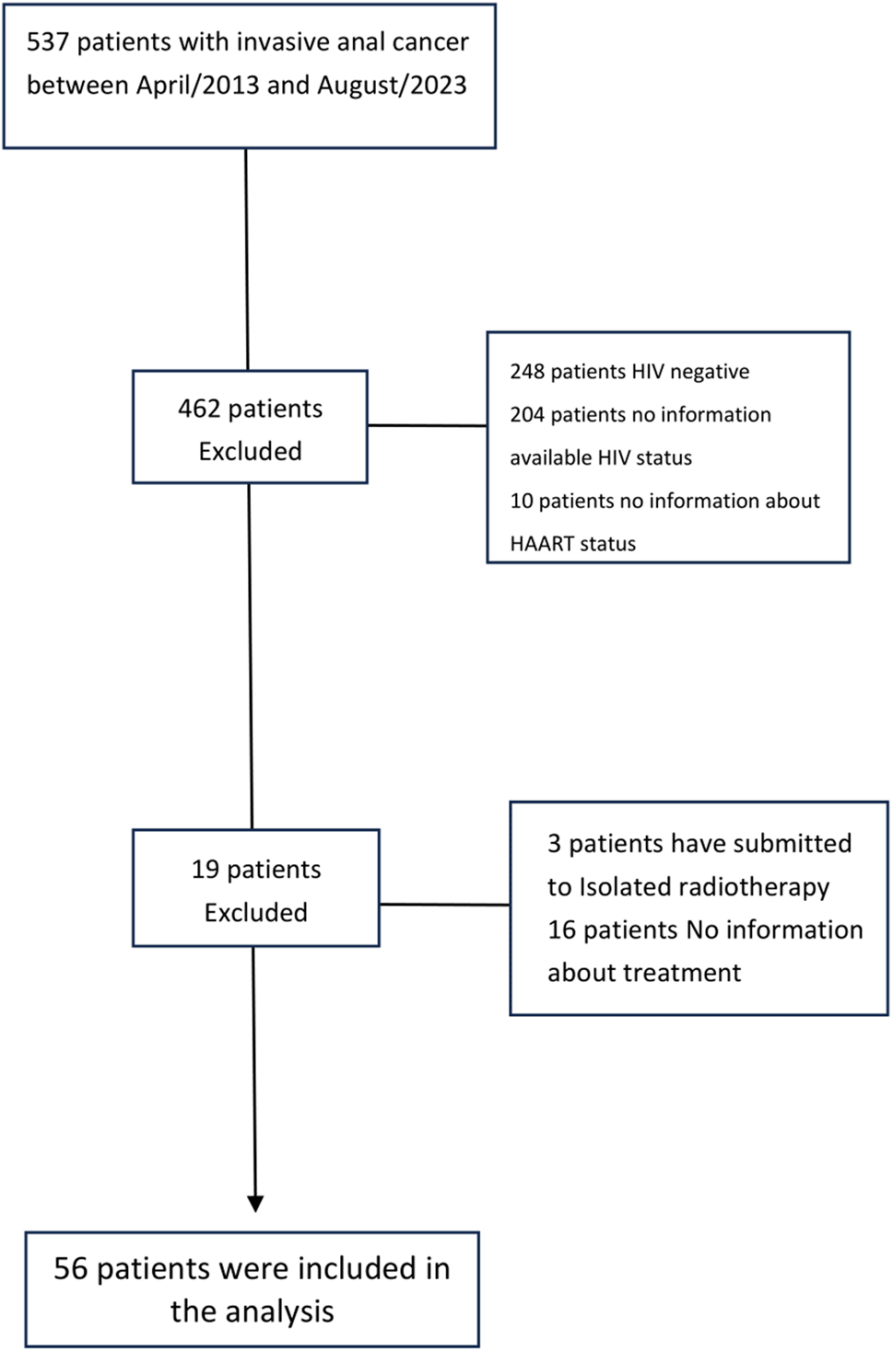
Flow diagram of patients screened for anal carcinoma, HIV-positive, and chemoradiotherapy

This cohort has evolved 537 patients with invasive anal carcinoma in stages I– III treated from April 2010 to August 2022 at Instituto do Câncer do Estado de São Paulo, Brazil. However, after applying inclusion and exclusion criteria, only 56 participants with invasive squamous cell carcinoma were considered for analysis due to being HIV-positive and having been submitted to definitive chemoradiotherapy (Fig. 1). Darunavir was the sulfonyl-HAART used more, followed by fosamprenavir. Most of the patients were male sex and white. The mean age was 52 and 53 years old for non-sulfonyl and sulfonyl-HAART, respectively. In terms of smoking status, no patient in the sulfonyl-HAART group was an actual smoker. Besides, none were overweight or obese. In terms of tumor characteristics, grade 2 of differentiation (moderate) was more prevalent. CD4 cell counts were initially assessed for normality using the Shapiro–Wilk test, with p-values of 0.01 for the non-sulfonyl group and 0.26 for the sulfonyl group, indicating a non-normal distribution in the former. A logarithmic transformation was subsequently applied; however, normality was still not achieved in the transformed data (*p* = 0.03 for the non-sulfonyl group and *p* = 0.46 for the sulfonyl group). Given the persistent deviation from normality, a non-parametric test was employed. Before the initiation of treatment, the median CD4 cell count was significantly higher in the non-sulfonyl group (468 cells/mm³) compared to the sulfonyl-HAART group (255 cells/mm³), with a statistically significant difference between groups (Mann–Whitney U test, *p* = 0.03) (Table 1).

### Global response rate

In both groups, the best response to chemoradiotherapy occurred six months after the treatment. The global response rate (complete response, partial response, stable disease or not available) occurred in 20 patients (48%) and 13 patients (100%) in non-sulfonyl-HAART and sulfonyl-HAART groups, respectively (Table 2). Association among CD4 count and type of HAART to response rate was assessed and summarized in Table 3.

**Table 1:** Characteristics of patients with invasive anal cancer treated with chemoradiotherapy

Variable	Non-sulfonyl-HAART N(%)	Sulfonyl-HAART N(%)	*p*-value
	42 (75%)	14 (25%)	
Sex			0.5^a^
Male	32 (76%)	9 (64%)	
Female	10 (24%)	5 (36%)	
Race			0.538^c^
White	34 (81%)	9 (64%)	
Latin	6 (14%)	3 (21%)	
Black	2 (5%)	2 (14%)	
Age(years)	52^⤉^ (± 8)	53^⤉^ (± 9)	0.6^e^
Smoking status			0.01^c^
Never	3 (7%)	3 (21%)	
Former	14 (33%)	0 (0%)	
Actual	9 (22%)	6 (43%)	
Missing	16 (38%)	5 (36%)	
BMI	24^⤉^ (± 4)	23^⤉^ (± 3)	0.6^b^
Missing	1	0	
Histologic differentiation			0.3^c^
Well	4 (10%)	2 (14%)	
Moderate	17 (40%)	8 (57%)	
Poor	4 (10%)	0	
Missing	17 (40%)	4 (29%)	
Tumor			0.11^c^
T1	0 (0%)	2 (15%)	
T2	15 (37%)	4 (31%)	
T3	12 (29%)	3 (23%)	
T4	14 (34%)	4 (31%)	
Node			0.24^c^
N0	16 (39%)	3 (23%)	
N1a	11 (27%)	4 (31%)	
N1b	4 (10%)	0 (0%)	
N1c	9 (22%)	6 (46%)	
Nx	1 (2%)	0 (0%)	
Staging			0.4^c^
I	1 (2%)	1 (8%)	
IIa	5 (12%)	1 (8%)	
IIb	7 (17%)	0 (0%)	
IIIa	9 (22%)	4 (31%)	
IIIb	4 (10%)	1 (8%)	
IIIc	15 (36%)	6 (46%)	
CD4 + cell count	^¥^468 (243–714)	^¥^255(217–338)	0.03^b^
ECOG performance scale			0.8^c^
0	15 (36%)	6 (43%)	
1	25 (60%)	7 (50%)	
3	2 (4%)	1 (7%)	
Type of Radiotherapy			0.7^c^
IMRT	18 (45%)	6 (50%)	
3D	11 (27.5%)	2 (17%)	
VMAT	11 (27.5%)	4 (33%)	
Toxicities grade 3 or 4			0.18^d^
No	17 (40%)	8 (62%)	
Yes	25 (60%)	5 (38%)	
Dose Chemo reduction			1.0^a^
No	38 (90%)	12 (92%)	
Yes	4 (10%)	1 (8%)	
Treatment discontinuation			0.7^a^
No	33 (83%)	10 (77%)	
Yes	7 (17%)	3 (23%)	
Response at 8 weeks			0.5^c^
Complete response	6 (14%)	4 (31%)	
Partial response	19 (45%)	3 (23%)	
Progression disease	2 (5%)	1 (8%)	
Missing	15 (36%)	5 (38%)	
Response at 6 months			0.001^c^
Complete response	16 (38%)	8 (61%)	
Partial response	2 (5%)	1 (8%)	
Progression of disease	9 (21%)	0 (0%)	
Not evaluate/Non-PD/SD	2 (5%)	4 (31%)	
Missing	13 (36%)	0 (%)	
Recurrence of disease			0.4^a^
No	22 (52%)	9 (69%)	
Yes	20 (48%)	4 (31%)	
Surgery rescue			1.0^a^
No	36 (86%)	12 (92%)	
Yes	6 (14%)	1 (8%)	

^a^Fisher’s exact; ^b^Mann-Witney test; ^c^Likelihood-ratio; ^d^Chi-square; ^e^T test; ^⤉^mean; ^¥^median (P25-P75); ^±^Standard deviation; BMI: Body Mass Index; PD: progression disease; SD: stable-disease

**Table 2:** Overall response rate to chemoradiotherapy in non-sulfonyl-HAART versus Sulfonyl-HAART groups

	Non-Sulfonyl-HAART N(%)	Sulfonyl-HAART N(%)	Odds ratio	CI 95%	p*
Response Rate			0	0–0.72	0.04
Global response**	20 (48%)	13 (100%)			
Progression disease	9 (21%)	0 (0%)			
Missing	13 (31%)	0 (0%)			

^*^2-side Fisher’s exact test. ^**^stable disease plus partial response plus complete response plus Not evaluate/Non-PD/SD

**Table 3:** Association between sulfonyl-HAART use and treatment response, with and without adjustment for CD4 count cells

Overall Response	OR (95% CI) ^**^	*p* ^*^
Unadjusted		
Sulfonyl-HAART	1	-
Adjusted for CD4 count cell		
Sulfonyl-HAART	1	-
CD4 count cell	1.00 (0.99–1.00)	0.3

*Logistic regression. **Odds ratio

### Progression-Free survival and overall survival outcomes

**Fig. 2:**
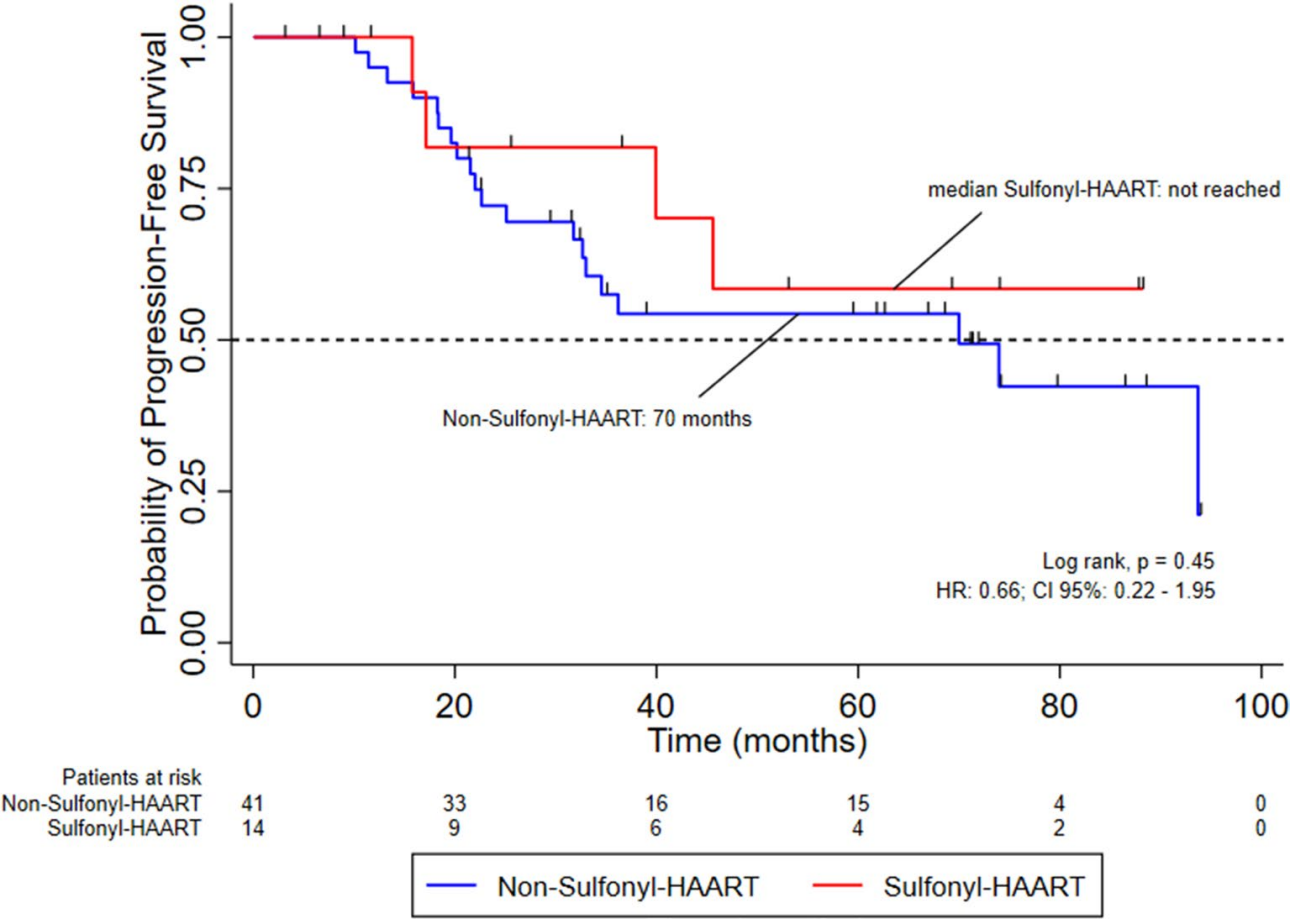
Progression-free survival probability of patients with early-stage anal cancer who are HIV-positive and treated with HAART containting sulfonyl-R during chemoradiotherapy

**Fig. 3:**
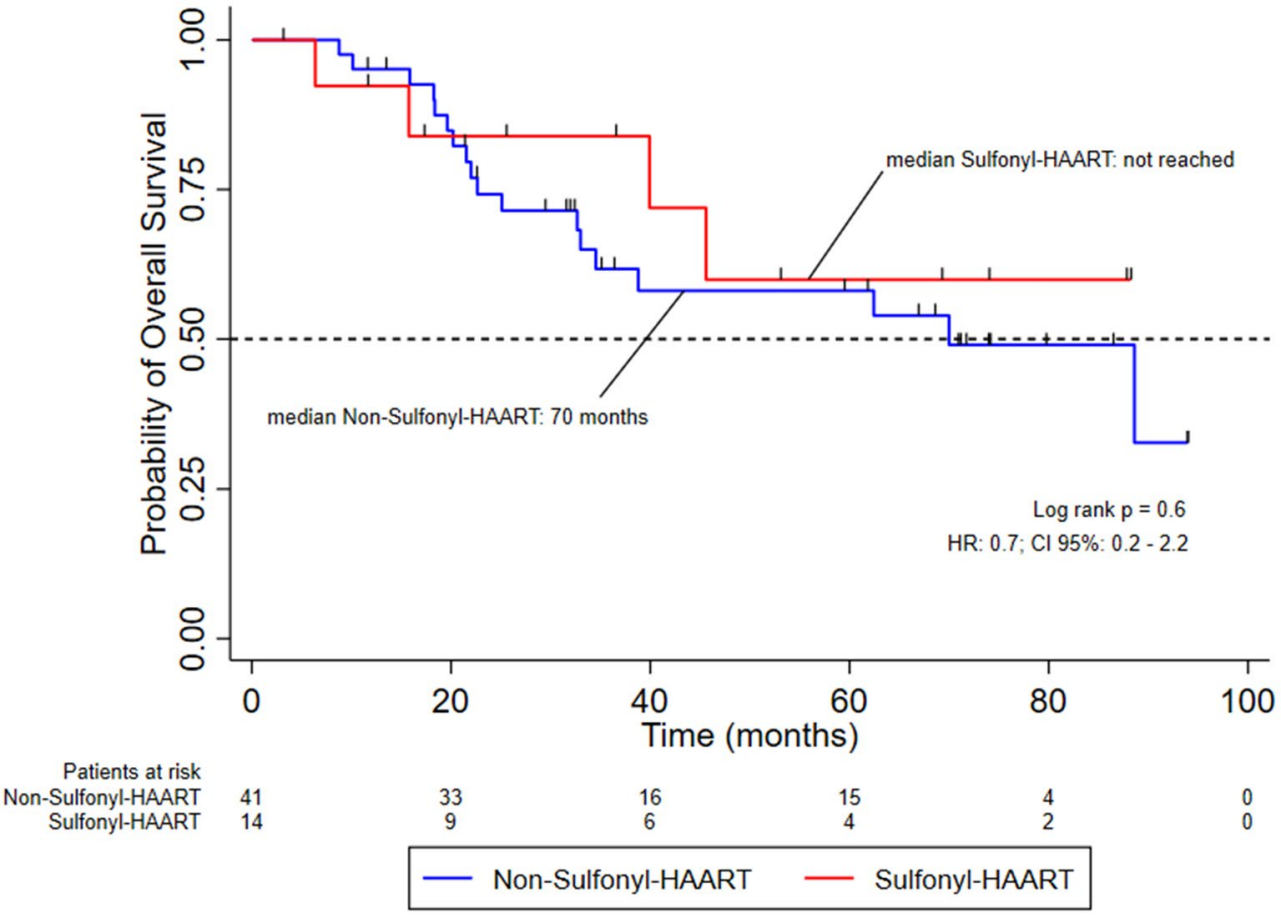
Overall survival probability of patients with early-stage invasive anal carcinoma who are HIV-positive and receiving HAART containting sulfonyl-R during chemoradiotherapy

Fifty-six patients HIV-positive with invasive anal cancer were included for analysis of survival in this cohort, divided between two groups: Non-sulfonyl-HAART and sulfonyl-HAART. The median follow-up was 60 months after the treatment of chemoradiation. Patients who received sulfonyl-HAART have a median of progression-free survival not reached, while for patients who received non-sulfonyl-HAART, the median was 70 months; Logrank, *p* = 0.6 (Fig. 2). In terms of overall survival, the numbers were the same for each group; Logrank, *p* = 0.45 (Fig. 3). Despite the overall survival numerically high for sulfonyl-HAART, these findings were not validated in a Cox multivariable regression modeling. Evaluating the association force of variables of interest to find risk factors and confounders for survival analysis, the hazard ratio (HR) for sulfonyl-HAART was 0.3 (*p* = 0.18). However, the HR for age, staging, and response at 6 months after treatment were 1.13, 1.8, and 1.51, respectively, and statistically meaningful (Table 4).

**Table 4:** Univariate and multivariate analysis of Cox proportional hazards

	Univariate analysis			Multivariate analysis*		
	HR	CI 95%	*p*	HR	CI 95%	*p*
Sex (Male/Female)	1.6	0.5–4.7	0.4	1.75	0.3–8.1	0.4
Age	0.9	0.9–1.0	0.9	1.13	1.0–1.2	0.032
Staging (I, II, III)	1.4	1.0–2.1	0.03	1.8	1.0–3.4	0.039
Dose QT reduction (N/Y)	0.9	0.2–4.1	0.9	-	-	-
Type of RT (IMRT, 3D, VMAT)	1.2	0.7–2.1	0.4	1.4	0.6–2.9	0.39
CD4 cell count	0.9	0.9–1.0	0.3	0.9	0.9–1.0	0.23
Discontinuation treatment by toxicity (N/Y)	1.9	0.7–4.9	0.1	1.5	0.3–6.1	0.52
Surgery rescue (N/Y)	2.0	0.7–5.6	0.1	1.39	0.2–7.5	0.69
Response at 8 w (N/Y)	1.4	1.0–1.8	0.009	1.33	0.9–1.9	0.12
Response at 6 mo (N/Y)	1.3	1.0–1.66	0.005	1.51	1.0–2.13	0.018
Sulfonyl-HAART (N/Y)	0.7	0.2–2.2	0.59	0.3	0.05–1.7	0.18

RT = radiotherapy; QT = chemotherapy; CR = complete response; PR = partial response; PD = progression of disease; HD = Hazard ratio; Y = yes; N = no. *Test of proportional-hazards assumption: *p* = 0.4 CI = Confidence Interval.

## Discussion

In this observational cohort, we found a higher overall response rate in patients with anal cancer HIV-positive who have taken HAART containing sulfonyl-R in its chemical composition during definitive chemoradiotherapy. When we tested the association adjusted to CD4 + cell count by logistic regression, we realized that it did not significantly impact the treatment response. In terms of survival outcomes, patients who took HAART-sulfonyl during chemoradiotherapy did not have better progression-free survival or overall survival. HAART-sulfonyl was a confounder in a multivariable Cox proportional hazard model. However, age, staging, and progression of disease at 6 months after treatment of chemoradiotherapy were configured as independent risk factors of lower survival. Each year more of age raised 13% the risk of death. Progression of disease at 6 months and locally advanced stage were associated with a rise in risk of 51% and 81% respectively.

Studies evaluating the association of type or class of HAART used during oncology treatment regimens and clinical outcomes such as treatment response rate and survival are unusual, despite some evidence suggesting antitumor activity, such as HAART containing sulfonyl-R. Most of this area’s research focuses on the toxicities of antiretroviral treatment concomitant to chemoradiotherapy. A study comparing 40 patients with anal cancer HIV-positive found a similar overall response rate and survival compared to HIV-negative patients, and high hematologic toxicities grade 3 and 4 in patients HIV-positive (33%) [13]. In another retrospective cohort, the authors reported that patients taking HAART during chemoradiotherapy had a high rate of hematologic toxicities grade 3 and 4, and radiotherapy interruption. However, neither study considered differences in the class of HAART or the presence of sulfonyl-R.

In 2008, Wexler and colleagues still reported that high viral burden and low CD4 count were associated with lower overall survival in 32 patients [14]. However, in the largest cohorts that evaluated CD4 count and survival outcomes in anal cancer patients with HIV-positive from 142 to 196 patients, they did not observe a negative impact on overall survival [15, 16]. In our study, despite the median CD4 count being significantly lower in the sulfonyl-HAART group, no impact on survival or response rate was verified.

Our study faced significant limitations due to substantial data loss. From an initial cohort of 537 patients, only slightly more than 10% were included in the analysis, primarily because 37% (n = 204) lacked HIV status information, resulting in reduced sample sizes for each group. Additionally, some variables had over 30% missing data, for example, the response rate in the non-sulfonyl-HAART group. In terms of global response, we observed a 100% (n = 13)response rate in the sulfonyl-HAART group; however, we interpreted this result cautiously due to the limited number of patients. When analyzing the association between HAART type and CD4 cell count with treatment response, we encountered a perfect drug effect, possibly indicating sampling bias, considering the small number of observations.

In summary, this observational study found that patients with invasive anal cancer HIV-positive in early stages taking HAART containting sulfonyl-R during chemoradiotherapy had a high overall response at 6 months after treatment. No difference was observed in terms of toxicities or survival. However, age and stagings advanced, as well as progression of disease at 6 months after treatment, were independent factors of poor survival. Despite the CD4 count being lower in the sulfonyl-HAART group, it was a confounder in survival outcomes and global response rate, according to our regression models. However, due to the retrospective nature of this study and the sampling size limitations, future studies should be conducted to confirm these results.

## Data Availability

No datasets were generated or analysed during the current study.
